# A Novel Model of Tumor-Infiltrating B Lymphocyte Specific RNA-Binding Protein-Related Genes With Potential Prognostic Value and Therapeutic Targets in Multiple Myeloma

**DOI:** 10.3389/fgene.2021.778715

**Published:** 2021-12-17

**Authors:** JingJing Zhang, Pengcheng He, Xiaoning Wang, Suhua Wei, Le Ma, Jing Zhao

**Affiliations:** ^1^ Department of Hematology, The First Affiliated Hospital of Xi’an Jiaotong University, Xi’an, China; ^2^ The First Affiliated Hospital of Xi’an Jiaotong University, Xi’an, China

**Keywords:** multiple myeloma, tumor-infiltrating B lymphocyte, RNA-binding protein, prognostic signature, immune-related signature, immunotherapy

## Abstract

**Background:** RNA-binding proteins (RBPs) act as important regulators in the progression of tumors. However, their role in the tumorigenesis and prognostic assessment in multiple myeloma (MM), a B-cell hematological cancer, remains elusive. Thus, the current study was designed to explore a novel prognostic B-cell-specific RBP signature and the underlying molecular mechanisms.

**Methods:** Data used in the current study were obtained from the Gene Expression Omnibus (GEO) database. Significantly upregulated RBPs in B cells were defined as B cell-specific RBPs. The biological functions of B-cell-specific RBPs were analyzed by the cluster Profiler package. Univariate and multivariate regressions were performed to identify robust prognostic B-cell specific RBP signatures, followed by the construction of the risk classification model. Gene set enrichment analysis (GSEA)-identified pathways were enriched in stratified groups. The microenvironment of the low- and high-risk groups was analyzed by single-sample GSEA (ssGSEA). Moreover, the correlations among the risk score and differentially expressed immune checkpoints or differentially distributed immune cells were calculated. The drug sensitivity of the low- and high-risk groups was assessed via Genomics of Drug Sensitivity in Cancer by the pRRophetic algorithm. In addition, we utilized a GEO dataset involving patients with MM receiving bortezomib therapy to estimate the treatment response between different groups.

**Results:** A total of 56 B-cell-specific RBPs were identified, which were mainly enriched in ribonucleoprotein complex biogenesis and the ribosome pathway. ADAR, FASTKD1 and SNRPD3 were identified as prognostic B-cell specific RBP signatures in MM. The risk model was constructed based on ADAR, FASTKD1 and SNRPD3. Receiver operating characteristic (ROC) curves revealed the good predictive capacity of the risk model. A nomogram based on the risk score and other independent prognostic factors exhibited excellent performance in predicting the overall survival of MM patients. GSEA showed enrichment of the Notch signaling pathway and mRNA cis-splicing via spliceosomes in the high-risk group. Moreover, we found that the infiltration of diverse immune cell subtypes and the expression of CD274, CD276, CTLA4 and VTCN1 were significantly different between the two groups. In addition, the IC50 values of 11 drugs were higher in the low-risk group. Patients in the low-risk group exhibited a higher complete response rate to bortezomib therapy.

**Conclusion:** Our study identified novel prognostic B-cell-specific RBP biomarkers in MM and constructed a unique risk model for predicting MM outcomes. Moreover, we explored the immune-related mechanisms of B cell-specific RBPs in regulating MM. Our findings could pave the way for developing novel therapeutic strategies to improve the prognosis of MM patients.

## 1 Introduction

Multiple myeloma (MM) is a B-cell hematological malignancy. The proliferation of plasma cells further induces end organ dysfunctions, including anemia, hypercalcemia, bone lesions and renal failure (Palumbo and Anderson, 2011). The incidence rate of MM has rapidly increased by 126% globally over the past 2decades (Cowan et al., 2018). The rapidly increasing incidence rate has underscored the urgent need for treatment improvement. Although the overall survival of multiple myeloma has been rapidly improved by the widespread application of stem cell transplantation and novel drugs represented by proteasome inhibitors and immunomodulatory drugs (Attal et al., 2017; Facon et al., 2019; Mikkilineni and Kochenderfer, 2021), MM remains incurable. The highly heterogeneous clinical outcomes of MM patients depend on the tumor burden, tumor cell characteristics, and especially genetic abnormalities. Currently, a risk classification model based on more detailed genetic and molecular information was created by the International Multiple Myeloma Working Group in 2015 (Palumbo et al., 2015). This staging system is widely used in clinical practice. Approximately 75% of patients who present without cytogenetic abnormalities are considered as low risk. These patients present heterogenous clinical outcomes (Binder et al., 2017). There remains a group of patients who are divided into low-risk groups characterized by therapy resistance, rapid refractory periods and short overall survival. Meanwhile, existing classification model fail to identify some of patients with 1q21 amplification and del 17p for very poor outcome. However, no attempts have been made to further sub-stratify such amount of patients (Walker et al., 2018). In light of the limitations of the current staging system, it is necessary to identify novel biomarkers and establish a prognostic model based on cytogenetic characterization to distinguish good prognosis from poor prognosis patients, thereby improving patients’ final prognosis.

The highly heterogeneous outcome of MM is mainly ascribed to the complex genomic landscape, including chromosomal gains or losses, structural variations, and cancer driver gene mutations (Manier et al., 2017). These genomic instabilities contribute to the clonal expansion of disease. As a result of the rapid development of high-throughput sequencing, posttranscript regulation (PTGR) has gained attention throughout the whole process of tumors (Gerstberger et al., 2014). RNA-binding proteins (RBPs) play key roles in posttranscript regulation by affecting gene expression and cellular metabolism (Yan et al., 2021). Studies have found that RBPs are functionally associated with tumor progression in different types of cancers, including multiple myeloma (Konishi et al., 2021; Wang et al., 2021).

The crucial role of the complex bone marrow microenvironment in MM progression and therapeutic response has been well established. The interactive relationship between tumor cells and the bone marrow environment is critical in promoting chromosomal instability in MM(Neuse et al., 2020). Single-cell RNA-sequencing datasets revealed in-depth interactions of stomal cells, myeloma cells and immune cells within the bone marrow microenvironment. These analyses found bone marrow mesenchymal stromal cells, accompanied by immune cells and aberrant genes involved in immune modulation and tumorigenesis (de Jong et al., 2021).

It has been gradually recognized that the success of chemotherapy and immunotherapy relies on the anticancer immune response (Fridman et al., 2017). The correlation between tumor-infiltrating lymphocytes and the clinical outcomes of cancers has been investigated (Fridman et al., 2012). The prognostic value of infiltrating T lymphocytes has been widely accepted. In contrast to T cells, the effects of infiltrating B cells in tumorigenesis and treatment are far from clear.

Growing evidence has indicated that Tumor-infiltrating B (TIL-B) cells contribute to the prognostic effect of tumors by inducing CD4^+^ T cells and CD8^+^ T cells, which help to regulate tumor invasion and metastasis (Wouters and Nelson, 2018).

Multiple myeloma is a plasmocytic disease. The core biological process of MM is genetic dysfunction throughout the multistep progression of B cell development (Pawlyn and Morgan, 2017). In the present study, we investigated the TIL-B-related RBP signature in MM. Furthermore, we propose a B-cell-specific RBP prognostic model of MM for the first time by combining immune, RBP and clinical characteristics. This model enables us to predict the clinical prognosis and therapeutic response of MM patients.

## 2 Materials and Methods

### 2.1 Patient and Tumor Cell Line Data Preparation

Transcriptional data of MM patients were downloaded from GSE24080, GSE4204 and GSE39754. GSE24080, including 559 newly diagnosed patients with MM(Mitchell et al., 2016), was used as the training set. GSE4204, including 538 newly diagnosed MM patients (Driscoll et al., 2010), was used as the validation set. These samples were analyzed on platforms GPL570, Affymetrix Human Genome U133 plus 2.0 array. GSE39754, including gene expression profiling of 170 newly diagnosed MM patients receiving bortezomib therapy (Chauhan et al., 2012), was used to compare the treatment response between different groups. A total of 1,542 RBPs were obtained from a previous study (Gerstberger et al., 2014). Expression data of RBPs in different cell types were downloaded from GSE42058 (4 samples of CD11c + cells), GSE49910 (4 samples of B cells, four samples of neutrophils, 24 samples of T cells, six samples of monocytes, eight samples of erythroblasts and a sample of bone marrow and progenitors), GSE51540 (9 samples of T cells), GSE59237 (10 samples of dendritic cells), GSE6863 (3 samples of dendritic cells), GSE8059 (3 samples of NK cells), GSE13906 (2 samples of gamma-delta T cells and two samples of lymphocytes), GSE23371 (12 samples of dendritic cells), GSE25320 (11 samples of mast cells), GSE27291 (12 samples of T cells), GSE27838 (16 samples of NK cells), GSE28490 (10 samples of monocytes, five samples of B cells, 10 samples of T cells, five samples of NK cells, four samples of eosinophils, five samples of mDCs, three samples of neutrophils and five samples of pDCs), GSE28726 (10 samples of NKT cells, eight samples of CD1d-aGC + Va24- T cells and eight samples of CD4 T cells), and GSE37750 (8 samples of plasmacytoid dendritic cells) and GSE39889 (16 samples of neutrophils). Each dataset was normalized, and all subsequent analyses were performed on normalized datasets.

### 2.2 Identification and Functional Analysis of Robust Prognostic B-Cell Specific RBP Signatures in MM

The Limma package (Ritchie et al., 2015) was used to screen differentially expressed RBPs among B cells and other cell types by following model: design < - model.matrix (∼group+0). Genes with FDR-corrected *p*-values below 0.01 were considered differentially expressed genes. Significantly upregulated RBPs in B cells were defined as B cell-specific RBPs. Gene ontology (GO) (Ashburner et al., 2000) and Kyoto Encyclopedia of Genes and Genomes (KEGG) (Kanehisa and Goto, 2000) enrichment analyses of B-cell-specific RBPs were applied by clusterProfiler in the R package (Yu et al., 2012; Wu et al., 2021). K-M analysis was performed to screen B-cell-specific RBPs associated with survival (*p* < 0.05). Then, univariate and multivariate Cox regressions were performed to further obtain a robust prognostic B-cell-specific RBP signature in MM (*p* < 0.05).

### 2.3 Construction of the Risk Model and Nomogram

The calculation formula for the risk score was defined as follows:

ExpGene1*Coef1 + ExpGene2*Coef2+ ExpGene3*Coef3.where Coef indicates the regression coefficients of genes, and Exp is the normalized expression value of each prognostic B cell-specific RBP signature. According to the median value of the risk score, MM patients in the training set were grouped according to the value of the risk score. K-M analysis was performed to identify the overall survival of all risk groups. ROC curves were plotted to evaluate the effectiveness of the risk model using the “survivalROC” routine in the R package. The risk model was tested in the validation set. Thereafter, Cox regression analyses were performed to identify independent prognostic factors for MM patients. The risk predictive model was plotted as a nomogram based on independent prognostic factors. The performance of the nomogram was evaluated by calibration and decision curves.

### 2.4 Immune Microenvironment of MM Patients in High- and Low-Risk Groups

Twenty-nine immune-related gene sets were used to perform ssGSEA (Subramanian et al., 2005) to calculate the enrichment infiltration of immune cells, pathways or functions in the MM samples. The 29 gene sets represented all types of subtypes of immune cells, potential functions, and related pathways described in a previous study (He Y. et al., 2018). Moreover, the correlations between the risk score and differentially enriched immune cells, pathways or functions and the correlations between the prognostic B-cell specific RBP signature and differentially enriched immune cells, pathways or functions were calculated. At the same time, the expression of immune checkpoints, including CD274 (also named PD-L1), CD276, CTLA4, PDCD1 and VTCNA, was compared between different groups. Additionally, the correlations between the risk score and differentially expressed immune checkpoints were calculated. Correlations were evaluated using Pearson tests.

### 2.5 Observation of Chemotherapeutic Response in Different Risk Groups

The IC50 values of 20 common chemotherapeutic drugs in the low- and high-risk groups were calculated by the pRRophetic algorithm via the GDSC database (Yang et al., 2013; Geeleher et al., 2014), while the percentages of complete response (CR), very good partial response (VGPR), no response, progression-free (NR) or progression and no response (Prog) were calculated to evaluate the treatment response to bortezomib therapy in the low- and high-risk groups.

### 2.6 Statistical Analysis

All of the data were analyzed by R software (version 4.0.0). Comparisons between low- and high-risk groups were calculated using Wilcoxon’s test.

## 3 Results

### 3.1 Identification and Functional Analysis of 56B-cell-specific RBPs

**FIGURE 1:**
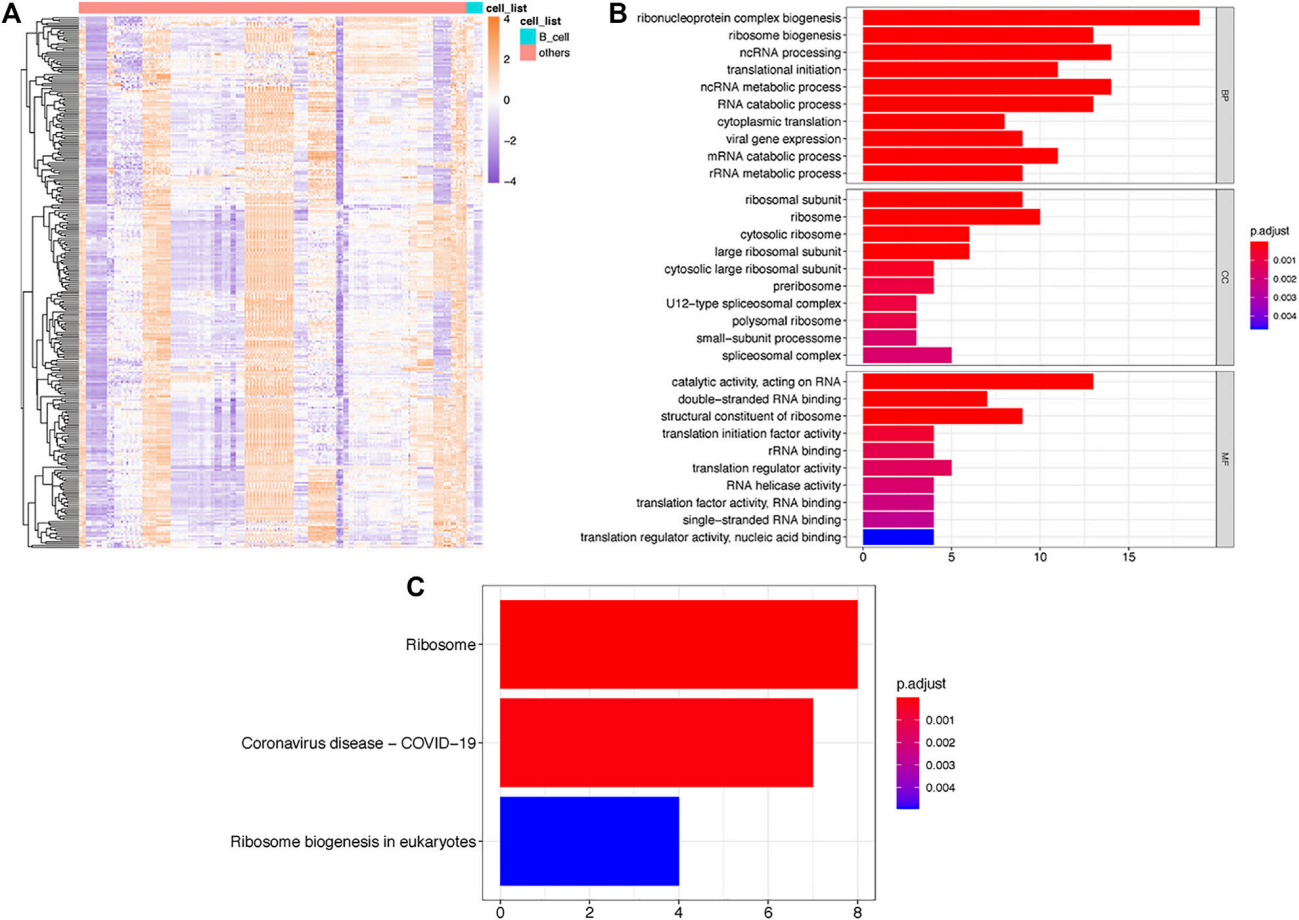
Identification of TILB-RBP related mRNAs. **(A)**. Heatmap of differential RPB gene expression in B cells and other immune cells. **(B)**. GO enrichment analysis of TILB-RBP-related mRNAs. **(C)**. KEGG pathway of TILB-RBPs-related mRNAs.

By comparing the expression of RBPs among B cells and other cell types, we found a total of 56 significantly upregulated RBPs (*p* < 0.01) and defined them as B cell-specific RBPs. Heatmap displaying differential gene expression in B cells and other cells (Figure 1A). GO enrichment analysis indicated that these B cell-specific RBPs were enriched in ribosome-related and RNA metabolism- and catabolism-associated BP, CC and MF, including ribonucleoprotein complex biogenesis, ribosome biogenesis, mRNA catabolic process, ribosomal subunit and catalytic activity, and acted on RNA. The top 10 BP, CC and MF are shown in Figure 1B. Similar to the GO results, we found that these B cell-specific RBPs were significantly enriched in KEGG pathways of ribosome and ribosome biogenesis in eukaryotes (Figure 1C).

### 3.2 Identification of ADAR, FASTKD1 and SNRPD3 as Prognostic Signatures in MM

#### 3.2.1 Purified Immune Cell Data

**FIGURE 2:**
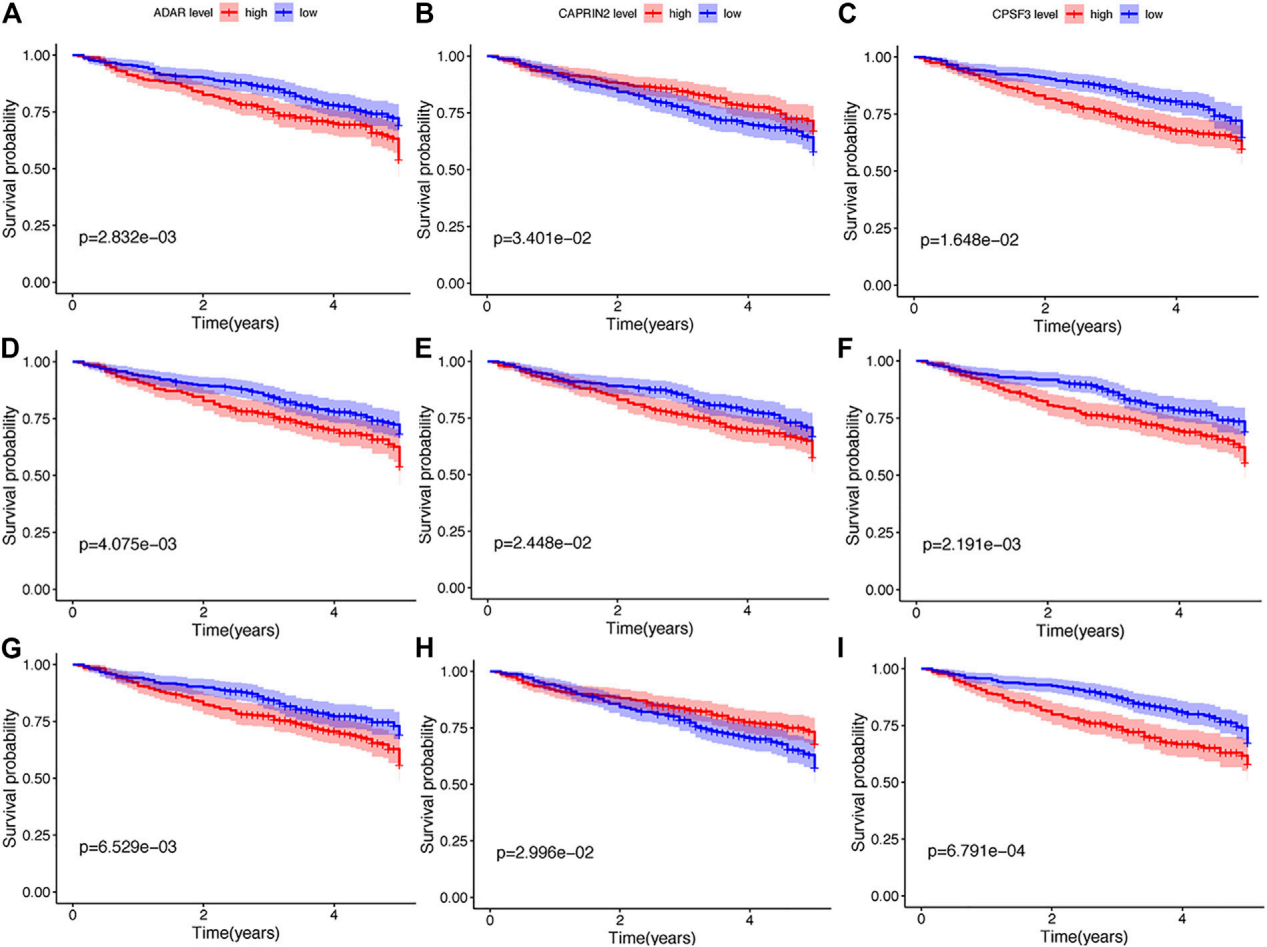
K-M analysis of nine differential genes regarding survival. **(A–G)**. Kaplan-Meier survival curves of Multiple myeloma with different expression levels of ADAR, CAPRIN2, CPSF3, DDX21, FASKD1, DROSHA, MRPL3, SART1, and SNRPD3.

**FIGURE 3:**
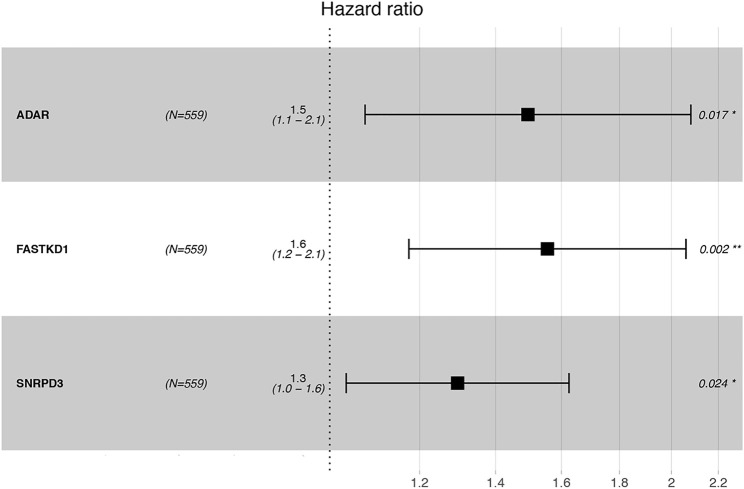
Forest plot visualizing the HRs of univariate Cox analysis of the TILB-RBPs and prognosis.

Thereafter, we investigated the prognostic value of these B cell-specific RBPs. First, according to the expression of each RBP, we divided MM patients into low- and high-RBP expression groups. K-M analysis revealed that patients in the groups with lower expression of FASKD1, SNRPD3, DDX21, MRPL3, ADAR, CPSF3, DROSHA, and CAPRIN2 and higher expression of SART1 had better survival (Figure 2), suggesting that FASKD1, SNRPD3, DDX21, MRPL3, ADAR, CPSF3, DROSHA, CAPRIN2 and SART1 might play important roles in the prognosis of MM patients. Next, univariate Cox regression analysis showed that FASKD1, SNPPD3, DDX21, MRPL3, ADAR, CPSF3 and DROSHA were closely related to the outcomes of patients (*p* < 0.05, Table 1). To further obtain a robust prognostic signature, we performed multivariate Cox regression algorithm analysis and found that ADAR, FASKD1 and SNRPD3 were significantly correlated with prognosis (*p* < 0.05, Table 2), and all of them acted as risk factors (HR > 1, Figure 3). Thus, ADAR, FASKD1 and SNRPD3 were identified as prognostic B cell-specific RBP signatures in MM and were used for subsequent construction of the risk model.

**TABLE 1:** Univariate Cox regression analysis results of differential RBPs.

Gene	Hazard ratios	CL95	*p*-value
ADAR	1.76	1.28–2.41	0.000
CPSF3	1.83	1.29–2.60	0.001
DDX21	1.58	1.17–2.11	0.002
DROSHA	1.64	1.12–2.39	0.010
FASTKD1	1.74	1.32–2.31	0.000
MRPL3	1.73	1.18–2.54	0.001
SNRPD3	1.48	1.19–1.84	0.000
CAPRIN2	0.82	0.58–1.16	0.262
SART1	0.79	0.58–1.06	0.118

**TABLE 2:** Multivariate Cox regression algorithm analysis of ADAR, FASKD1 and SNRPD3 gene expression with prognosis.

Gene	Coef	HR	HR.95L	HR.95H	*p*-value
ADAR	0.40216328	1.49505543	1.07431169	2.08057937	0.01707585
FASTKD1	0.44163922	1.55525453	1.17418704	2.05999263	0.00207,193
SNRPD3	0.25941536	1.29617207	1.03397688	1.62485454	0.02446633

#### 3.2.2 Construction of the Risk Score Model and Nomogram Based on the Prognostic B-Cell Specific RBP Signature in MM

**FIGURE 4:**
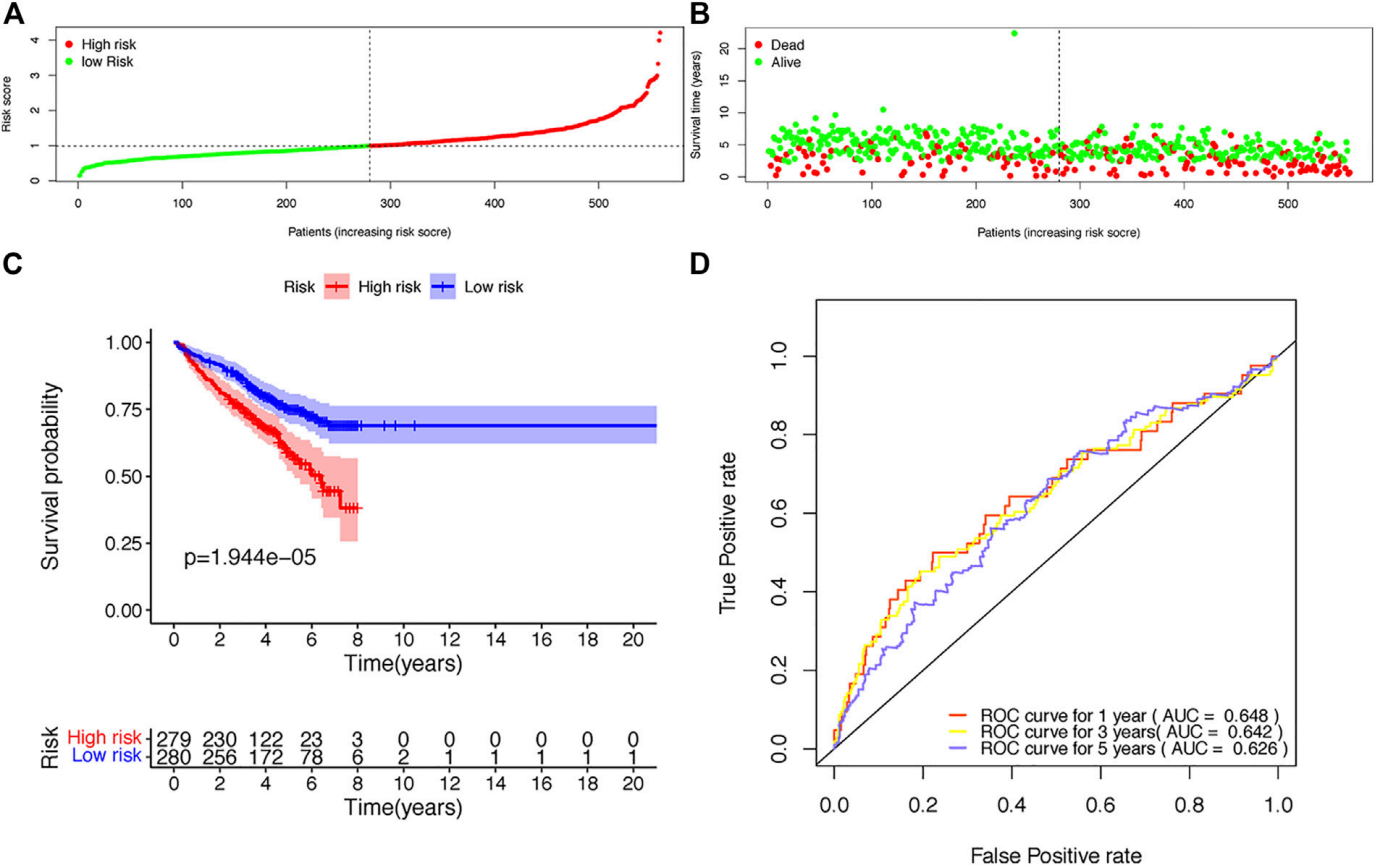
Construction of the risk score model based on the prognostic B-cell specific RBP signature in MM. **(A)**. Patient distribution by different risk scores in the training set. **(B)**. Survival status of all patients in the training set. **(C)**. Kaplan-Meier survival curves of patients in the high-risk and low-risk groups. **(D)**. ROC curve analysis according to the 1–5 years survival of the area under the ROC curve value in the training set.

**FIGURE 5:**
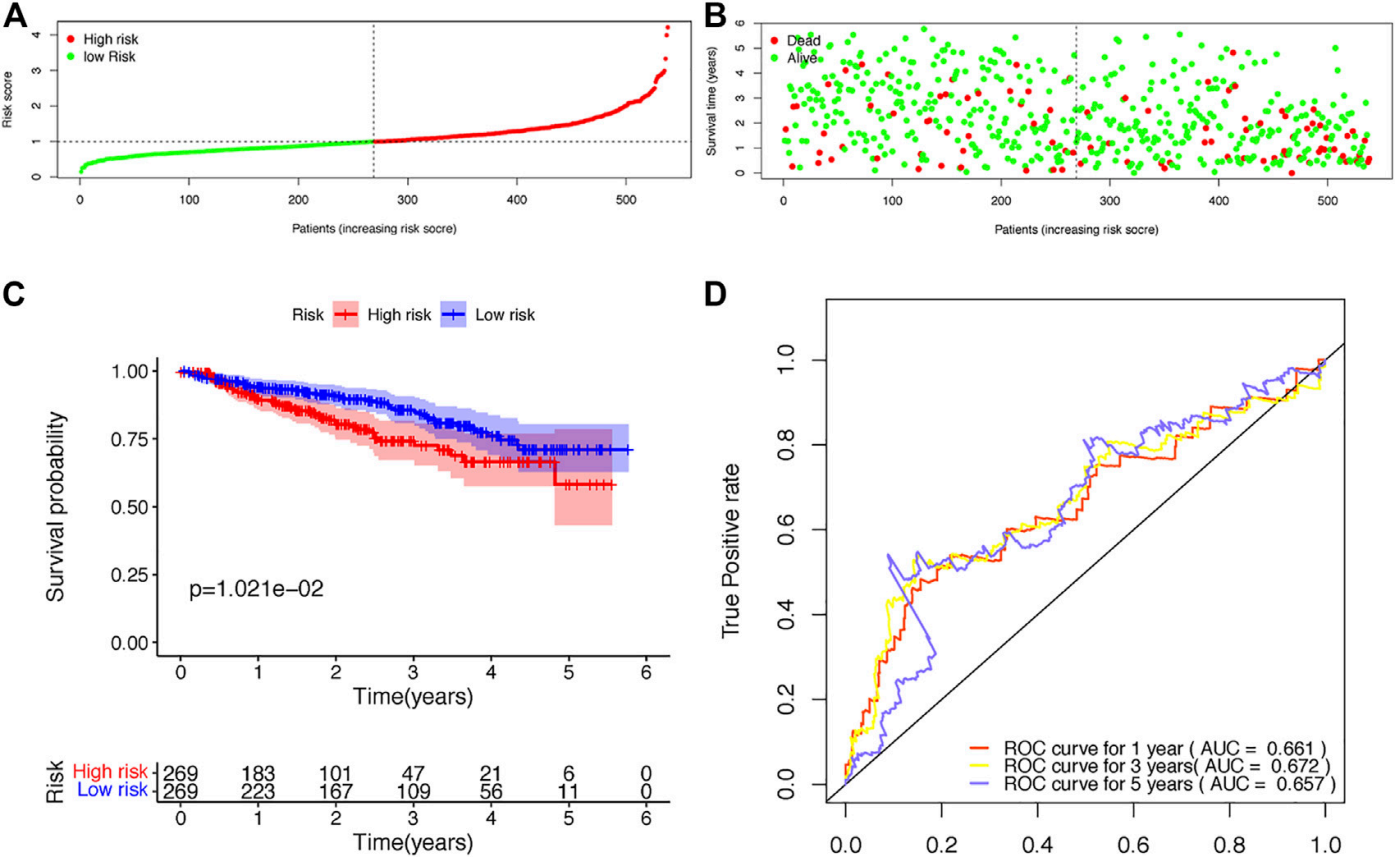
Validation of the risk score model based on the prognostic B-cell specific RBP signature in MM. **(A)**. Patient distribution by different risk scores in the validation set. **(B)**. Survival status of all patients in the validation set. **(C)**. Kaplan-Meier survival curves of patients in the high-risk and low-risk groups. **(D)**. ROC curve analysis according to the 1–5 years survival of the area under the ROC curve value in the validation set.

The risk scores of each patient were calculated according to the expression levels and coefficients of ADAR, FASKD1 and SNRPD3. Patients in the training set were divided into high- and low-risk groups according to the median risk score (Figure 4A). The distribution of all patients’ survival status in the training set is shown in Figure 4B. Patients in the low-risk group had a survival advantage over patients in the high-risk group (Figure 4C). The areas under the ROC curves for 1-,3–5 years survival were 0.648, 0.642 and 0.626, respectively, suggesting good performance of the risk model in the training set (Figure 4D). The risk model was further tested in the validation set, and similar results were obtained (Figures 5A–D).

**FIGURE 6:**
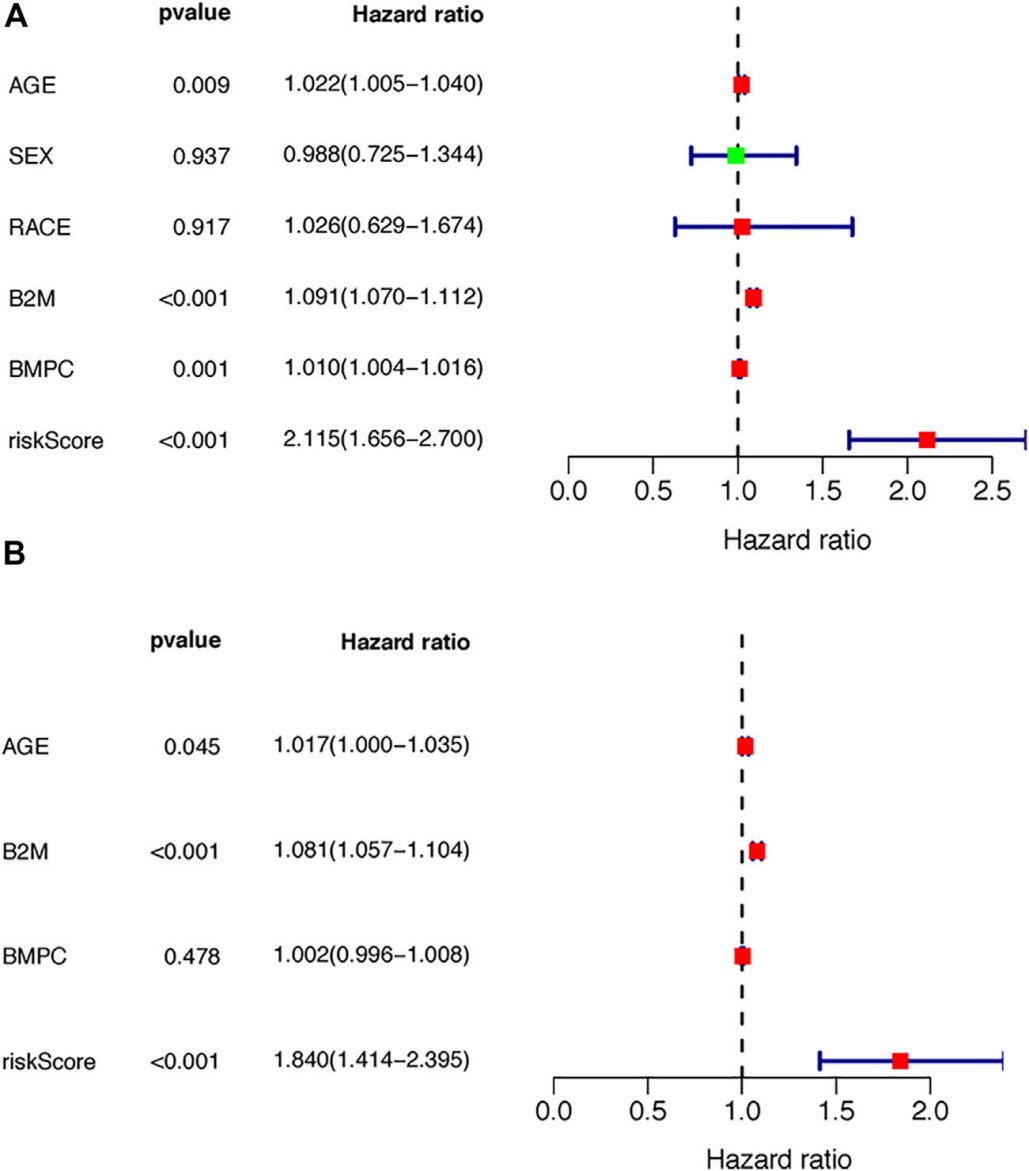
Independence of the TILB-RBPs. A. Forest plot visualizing the HRs of univariate Cox analysis of the TILBlncSig and clinicopathological factors in **(A)** the TCGA discovery dataset **(B)** the TCGA testing dataset; and **(C)** the GSE31684 dataset.

Next, we conducted univariate and multivariate analyses to detect independent prognostic factors. The univariate results showed that age, B2M, BMPC and risk score were significantly associated with the overall survival of MM patients (Figure 6A). Age, B2M, BMPC and risk score were then included in the multivariate analysis, and we found that the risk score was remarkably related to prognosis (Figure 6B), indicating that the risk score was an independent prognostic factor for poor prognosis in MM.

**FIGURE 7:**
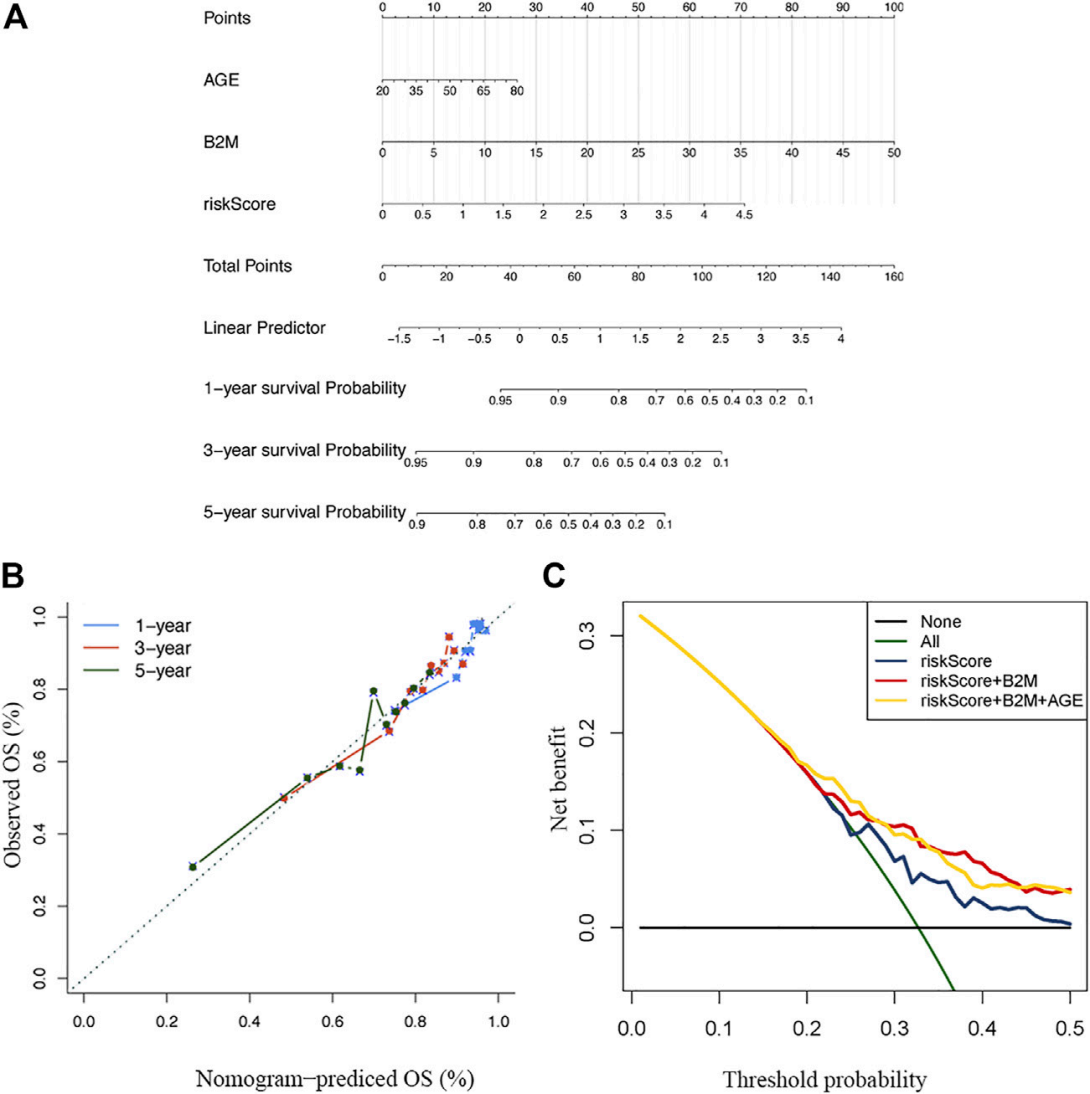
Construction and verification of the nomogram **(A)** A nomogram combining clinical signatures and prognostic factors to predict the 1–5 years survival rate of MM patients **(B)** The 5 years calibration chart verifies the predictive ability of the nomogram **(C)** The 5 years decision curve analysis of the clinical benefit rate.

Thereafter, we constructed a nomogram with a C-index of 0.667 to predict the 1-, 3–5 years survival of MM patients, combined with independent prognostic factors (age, B2M and risk score) obtained by the above multivariate analysis (Figure 7A). The slopes of the calibration curves for 1-, 3–5 years survival were close to 1 (Figure 7B), indicating the high accuracy of the nomogram. In addition, the decision curves, which displayed the clinical utility of each model, indicated that the nomogram had better survival prediction performance than the risk model (Figure 7C).

### 3.3 Functional Analysis of Prognostic B-cell-specific RBP Genes by GSEA

**FIGURE 8:**
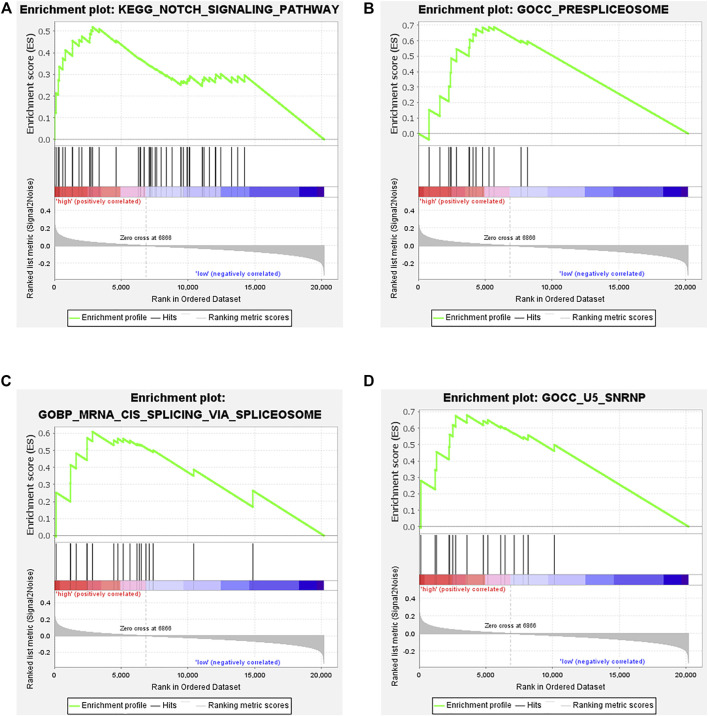
Functional analysis of genes in the low- and high-risk groups by GSEA. **(A)**. Notch signaling pathway, **(B)**. prespliceosome, **(C)**. mRNA cis-splicing via spliceosome, **(D)**. U5 snRNP.

To better understand the underlying mechanisms of the prognostic B cell-specific RBP signature in regulating MM, we first analyzed the functions of genes by GSEA. We found that the Notch signaling pathway, prespliceosome, mRNA cis-splicing via spliceosome and U5 snRNP were notably enriched in the high-risk group (*p* < 0.01), while olfactory receptor activity, sensory perception of smell, response to amphetamine, establishment of pigment granule localization, regulation of renal system process, pigment granule localization, olfactory transduction, mating, and odorant binding were enriched in the low-risk group (*p* < 0.01, Figure 8).

### 3.4 Immune Microenvironment of Low- and High-Risk Groups

**FIGURE 9:**
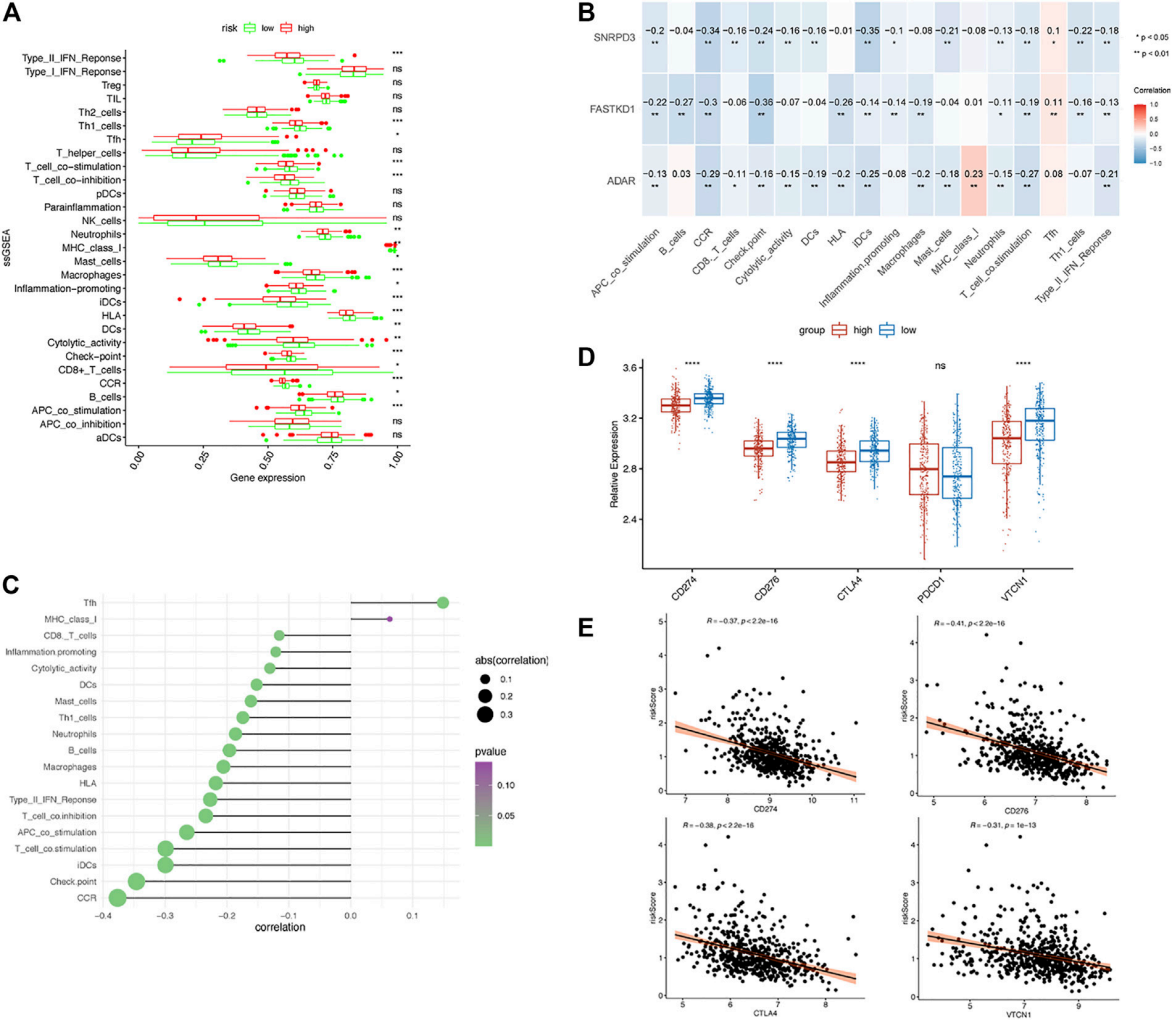
Immune microenvironment of the low- and high-risk groups. **(A)**. Boxplots of the immune cell infiltration cluster in the high- (red)- and low- (green)-risk groups stratified by the TILB RBP prognostic model. **(B)**. Correlation between the expression of ADAR, SNRPD3 and FASTKD1 and the immune cell infiltration cluster. **(C)**. Correlation between the risk score and immune cell infiltration cluster. **(D)**. The differential expression of immune checkpoints, including CD274, CD276, CTLA4 and VTCN1, in the low-risk and high-risk groups. **(E)**. Association between the risk score and the expression of CD274, CD276, CTLA4 and VTCN1.

Next, we performed ssGSEA to detect the enriched distribution of different immune cells, pathways or functions. We analyzed the expression of immune checkpoints further to evaluate the immune microenvironment differences between the two groups. The ssGSEA results showed that the enrichment level of Tfhs was lower in the low-risk group, and other immune cells, including B cells, CD8^+^ T cells, T cell coinhibition, T cell costimulation, Th1 cells and type II IFN response, DCs, iDCs, APC costimulation, CCR, checkpoint, HLA, inflammation-promoting, macrophages, mast cells, MHC class Ⅰ, and neutrophils, were more highly enriched in the low-risk group (Figure 9A). All of the enrichment levels of the immune cells, except Tfh and MHC class I, were negatively correlated with the expression of ADAR, FASKD1 and SNRPD3 (*p* < 0.05, Figure 9B). We also found that the risk score was negatively correlated with enrichment levels of the immune cells (*p* < 0.05, Figure 9C). Consistent with the ssGSEA results, we observed that the expression of immune checkpoints, including CD274, CD276, CTLA4 and VTCN1, was remarkably higher in the low-risk group (*p* < 0.01, Figure 9D), while the risk score was negatively correlated with the expression of CD274, CD276, CTLA4 and VTCN1 (*p* < 0.01, Figure 9E). All of these results suggested that the prognostic B-cell-specific RBP signature influenced the immune microenvironment of MM patients, and a higher risk score could indicate lower antitumor immunity in MM patients.

### 3.5 Validation of the Prognostic Value of TBIL-RBPs in the Chemotherapeutic Response of MM

**FIGURE 10:**
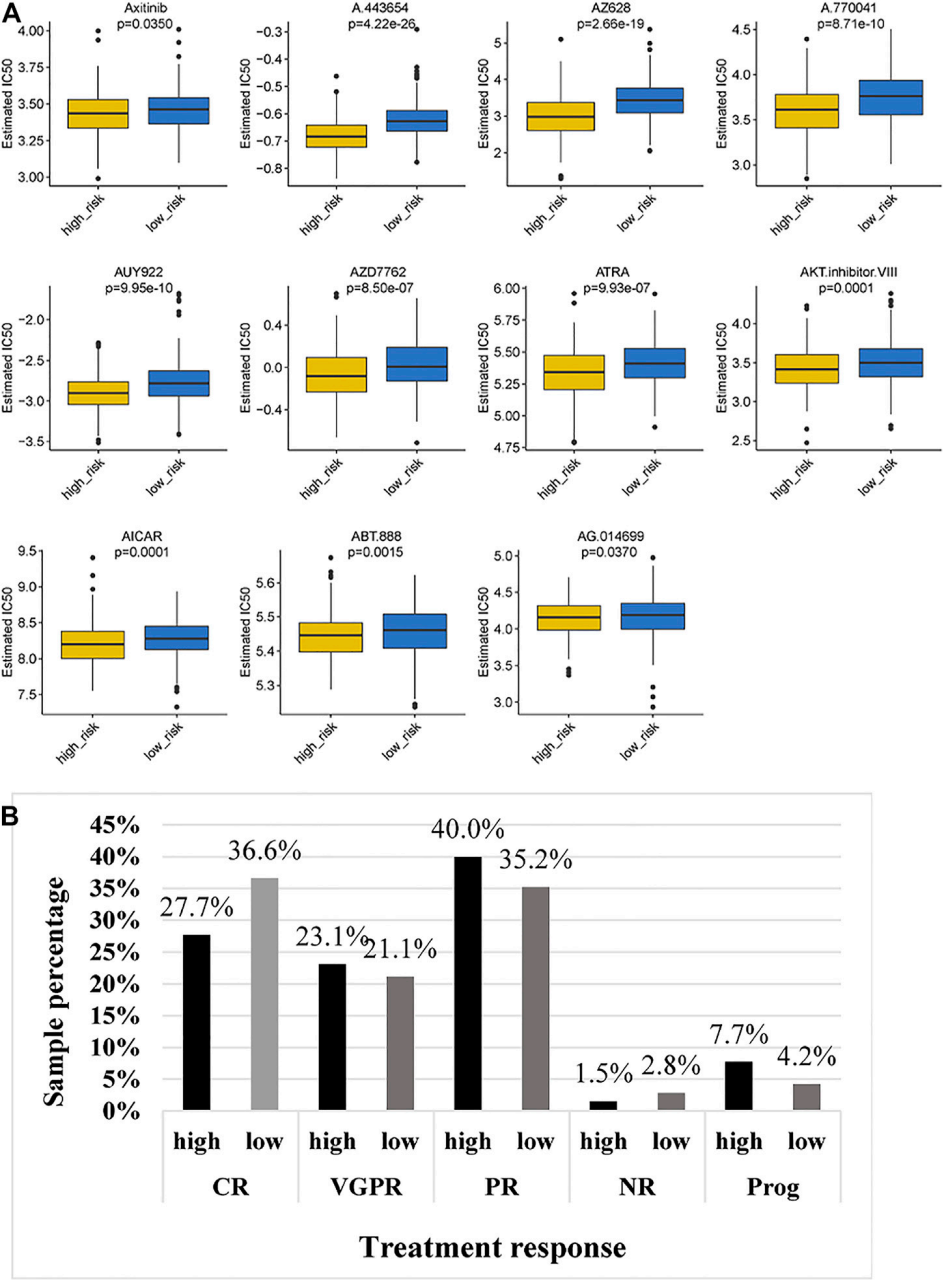
Chemotherapeutic response of MM patients in the low- and high-risk groups. **(A)**. Comparison of IC50 of chemotherapeutic drugs between the high-risk and low-risk groups. **(B)**. Bortezomib treatment response of MM patients in the high-risk and low-risk groups.

Given the different immune microenvironments between the low- and high-risk groups, we hypothesized that the response to drugs might be different between the two groups. The IC50 of A.443,654, A.770,041, ABT.888, AG.014699, AICAR, AKT. inhibitors VIII, ATRA, AUY922, axitinib, AZ628 and AZD7762 were significantly higher in the low-risk group (Figure 10A), indicating that patients in the low-risk group were more sensitive to these drugs. In addition, we compared the treatment response to bortezomib therapy in different risk classification groups. We found that a larger proportion of patients (36.6%) in the low-risk group had CR to bortezomib therapy than that (27.7%) in the high-risk group (Figure 10B), suggesting that the TBIL-RBPs might be a potential biomarker of bortezomib treatment response for MM patients.

## 4 Discussion

Multiple myeloma is a B cell hematological malignancy with insidious onset. Once diagnosed, most patients suffer from multiorgan dysfunction. The incidence rate of MM has increased rapidly over the last decade. Substantial strides have been made in the treatment of MM. However, for some reasons, including a lack of early detection and complex cytogenetic abnormalities, the majority of MM patients continue to relapse, and a minority of MM patients even suffer from early relapse and resistance to chemotherapy and immunotherapy, gaining little benefit from advances in therapy (Kumar et al., 2017). The application of genomic technologies has led to a better understanding of the underlying biology of MM(Lohr et al., 2014). At the same time, it is widely accepted that dysregulated immunological processes in the tumor microenvironment are closely related to the progression of MM(Nakamura et al., 2020; Botta et al., 2021). Thus, we concentrated on the cytogenetic heterogeneity of MM and the correlation between tumor immune cell infiltration and tumor cells. Using RNA sequencing data and clinical data in GEO, we constructed a novel prognostic model based on B cell-specific RBP-associated genes, which are of remarkable importance in the early diagnosis, prognosis prediction and therapeutic evaluation. Subsequently, we verified the predictive value of the model in validation datasets. Furthermore, a nomogram with high accuracy for predicting the overall survival of MM patients was constructed based on the TBIL-RBPs and other independent prognostic factors, as evidenced by calibration and decision curves.

In the present study, we first conducted a comparison analysis among different immune cell lines. Fifty-six highly specifically expressed TILB-RBPs were preferentially observed in B cell lines compared with other immune cell lines. Furthermore, functional enrichment analysis revealed that these B cell-specific RBPs were closely related to the immune response, ribosome biogenesis and RNA metabolism. RBPs play key roles in posttranscriptional regulation via genetic changes, epigenetic alterations, and noncoding RNA mediation, which are essential in the malignant transformation of cancers (Bitaraf et al., 2021). RBPs are also essential in tumorigenesis in hematological malignancies. Insulin-like growth factor 2 mRNA binding proteins (IGF2BPs) are described as major regulators of stem cells. IGF2BP1 and IGF2BP3 are overexpressed in translocation-ETV6/RUNX1-positive B-ALL (Elcheva et al., 2020). Acute myelocytic leukemia patients with high expression of IGF2BP2 had worse overall survival (He X. et al., 2018). Musashi-2 protein (MSI2) is overexpressed in acute myeloid leukemia (AML) cell lines, and high expression of MSI2 promotes proliferation and inhibits apoptosis of AML cells. High expression of MSI2 in AML patients correlates with poorer survival in patients, thereby defining MSI2 as a prognostic biomarker for therapy in AML (Kharas et al., 2010). There have also been several reports of specific low-occurrence mutations in RPL5 and RPL10 and overexpression of RPS9 in MM that were closely related to tumorigenesis and clinical outcomes (Dabbah et al., 2021). These studies were in accordance with our findings that RBPs could be potential prognostic biomarkers.

To further define the role of the TILB-RBPs in the clinical outcomes of MM, the relationship between TILB-RBPs and overall survival was assessed. We identified 3B cell-specific RBP genes -- ADAR, FASTKD1 and SNRPD3—which were significantly correlated with the outcomes of MM patients. ADAR-mediated A-to-I editing is a key form of posttranscriptional regulation in human physiology (Vesely and Jantsch, 2021). ADAR1 is the most abundant and active RNA editing enzyme in MM and is recognized as an oncogenic central driver of cancer cell proliferation (Teoh et al., 2018). ADAR1 promotes malignant regeneration of MM by mediating the recoding of the self-renewal agonist GLI1, which activates the Hh pathway and promotes the production of cancer stem cells (Lazzari et al., 2017). FASTK family proteins have been verified to be linked to mitochondrial diseases by regulating mitochondrial RNA homeostasis (Boehm et al., 2017). Some studies have confirmed that FASTKD1 is related to the occurrence of tumors. For example, FASTKD1 was associated with poor prognosis of ALL in children and adults (Wang et al., 2015). FASTKD1 could also be used as a biomarker of primary endometrial tumors (Colas et al., 2011). SNRPD3, also called SMD3, binds to small nuclear RNA to affect the formation of small nuclear ribonucleoprotein particles (Camasses et al., 1998). Studies have revealed that silencing of SNRPD3 causes overexpression of p53 levels, thereby modulating CDKN1A expression and further influencing the cell cycle arrest and cell death of NSCLC cells (Siebring-van Olst et al., 2017). In addition, a study also found that SNRPD3 might be a novel breast cancer-related biomarker (Zhang et al., 2015). In our study, we found for the first time that FASTKD1 and SNRPD3 are related to the prognosis of multiple myeloma, and the specific function and mechanism of these genes in tumorigenesis in multiple myeloma require further study. At the same time, we calculated the risk score and constructed a predictive model based on these three genes. The results of the ROC curve analysis showed that the model has good predictive effects. In addition, univariate and multivariate regression analyses indicated that the risk score was an independent prognostic factor. At the same time, a nomogram was constructed for predicting the survival of patients with multiple myeloma at 1, 3 and 5 years. The C index and correction curve of the nomogram showed that the prediction model has high prediction accuracy for 1, 3 and 5 years and has clinical value.

To further clarify the role of the TILB-RBPs in stratifying survival, the association of the TILB-RBPs and survival in MM was assessed. Patients were grouped based on the risk score. First, GSEA functional enrichment analysis was performed for all genes in different groups. The results revealed that the Notch signaling pathway and biological processes and cellular components related to RNA splicing were significantly enriched in the high-risk groups. The Notch pathway is crucial to cell cycle regulation. Accumulating evidence has shown that the Notch pathway deregulates MM in tumorigenesis and drug resistance, especially in proteasome inhibitor resistance (Colombo et al., 2013). Deregulation of Notch signaling in MM occurs throughout the pathogenesis of plasma cells (Saltarella et al., 2019). Notch receptors and their ligands affect not only MM cells but also bone marrow stroma to further regulate the adhesive behavior of MM (Nefedova et al., 2004). In addition, the Notch pathway plays a vital role in immune regulation by stimulating the proliferation of T regulatory cells and upregulating TGF-*β* receptor II to suppress antitumor T-cell responses (Hue et al., 2012). An increasing number of studies have shown that the RNA spliceosome pathway is a major factor in cancer progression. A study revealed that aberrant RNA splicing patterns were relevant to worse survival outcomes of MM patients, which could be used for the risk stratification of patients (Bauer et al., 2021). Moreover, a study showed that inhibition of the spliceosome could synergize with proteasome inhibitors to potentiate antitumor effects. This unreported mechanism of the spliceosome suggests that spliceosome targeting could serve as a potential therapeutic target in myeloma (Huang et al., 2020). The above results are in accordance with our findings that prognostic characteristic genes could affect the prognosis of patients with multiple myeloma by regulating the splicing of precursor mRNA, activation of the Notch pathway and RNase L and ribosomal nucleoprotein synthesis.

Subsequently, we also compared the immune microenvironment in different groups. We found that there were significant differences in immune cell infiltration, immune-related functions, immune-related pathways and the expression of immune checkpoint genes between the two groups. Several single-cell transcriptional studies have revealed that transcriptional programs are associated with aggressive myeloma progression and immune evasion (Ryu et al., 2020; Liu et al., 2021). According to the above findings, we present the hypotheses that the prognostic characteristic genes are highly associated with different immune microenvironments in the two groups. Subsequently, we conducted a correlation analysis of TBIL-RBPs and immune cell infiltration. We found that the expression of ADAR, FASTKD1 and SNRPD3 was negatively correlated with the infiltration, functions and pathways of immune cells. The risk score was also negatively correlated with the expression of immune checkpoints, indicating that ADAR, FASTKD1 and SNRPD3 might interact with the immune microenvironment of multiple myeloma. TBIL-RBPs might further influence the immune response of MM patients, response to treatment, and prognosis.

We finally analyzed the Genomics of Drug Sensitivity in Cancer (GDSC) dataset to further validate the prognostic effect of the risk score. The GDSC is a large dataset including cell viability and response to drugs (Yang et al., 2013). We found that the IC50 of 11 drugs in the low-risk group was significantly higher than that in the high-risk group, indicating that patients in the low-risk group might have greater sensitivity to these 11 drugs. Strikingly, the high-risk group presented less sensitivity to bortezomib treatment. These results, together with previous observations, supported the risk score based on TILB-RBPs and demonstrated good accuracy for prognostic assessment. The TILB-RBPs were shown to have prognostic value not only for chemotherapy but also for immunotherapy. Nonetheless, there are limitations of our current study. First the prognostic model still needs to be further validated in other independent large sample cohorts to ensure the reliability of the model before clinical use. In addition, more functional experiments *in vivo* and vitro are still needed to further reveal the possible mechanisms for TILB-RBPs.

## 5 Conclusion

In conclusion, in this study, we identified 3 B lymphocyte-specific RBPs significantly related to the overall survival of MM patients and further established a risk model based on these genes. The good predictive value of the model was verified in the validation set. Application of the TBIL-RBPs to immunotherapy datasets revealed that the risk model can assess not only chemotherapy but also immunotherapy response. To the best of our knowledge, our study is the first to investigate B lymphocyte specific RBPs in MM, emphasizing the impact of TILB-RBPs on clinical outcomes and treatment response. The results of this study could provide a basis for individualized precision therapy in the future. The three prognostic genes—ADAR, FASTKD1 and SNRPD3 -- could be potential new prognostic and therapeutic biomarkers of MM.

## Data Availability

Publicly available datasets were analyzed in this study. This data can be found here: GSE24080, GSE4204, and GSE39754.
